# Genetic markers cannot determine Jewish descent

**DOI:** 10.3389/fgene.2014.00462

**Published:** 2015-01-21

**Authors:** Raphael Falk

**Affiliations:** Department of Genetics, Program for History and Philosophy of Science, The Hebrew University of JerusalemJerusalem, Israel

**Keywords:** genetics of race, biology of the Jews, evolution at DNA-sequence level, Y-chromosome inheritance of Cohanim, Khazar origins of Ashkenazim, horizontal vs. vertical inheritance

## Abstract

Humans differentiate, classify, and discriminate: social interaction is a basic property of human Darwinian evolution. Presumably inherent differential physical as well as behavioral properties have always been criteria for identifying friend or foe. Yet, biological determinism is a relatively modern term, and *scientific racism* is, oddly enough, largely a consequence or a product of the Age of Enlightenment and the establishment of the notion of human equality. In recent decades ever-increasing efforts and ingenuity were invested in identifying Biblical Israelite *genotypic* common denominators by analysing an assortment of *phenotypes*, like facial patterns, blood types, diseases, DNA-sequences, and more. It becomes overwhelmingly clear that although Jews maintained detectable vertical genetic continuity along generations of socio-religious-cultural relationship, also intensive horizontal genetic relations were maintained both between Jewish communities and with the gentile surrounding. Thus, in spite of considerable consanguinity, there is no Jewish genotype to identify.

## The biologization of race

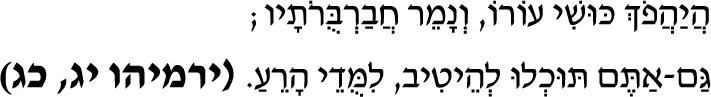

Can the Ethiopian change his skin, or the leopard his spots? Then may ye also do good, that are accustomed to do evil (Jeremiah 13, 23, *The Holy Bible*: King James Version, 2000).

A defining property of Darwinian evolution of humans is *social interaction*, and inherent differential physical as well as behavioral properties have always been the immediate criteria for identifying friend or foe. Skin color has been an obvious physical marker for the “other” and eventually a guide for socio-cultural interactions. Notwithstanding, the socio-cultural significance that humans gave to physical properties was only one of the widespread discriminating variables.

**Figure 1:**
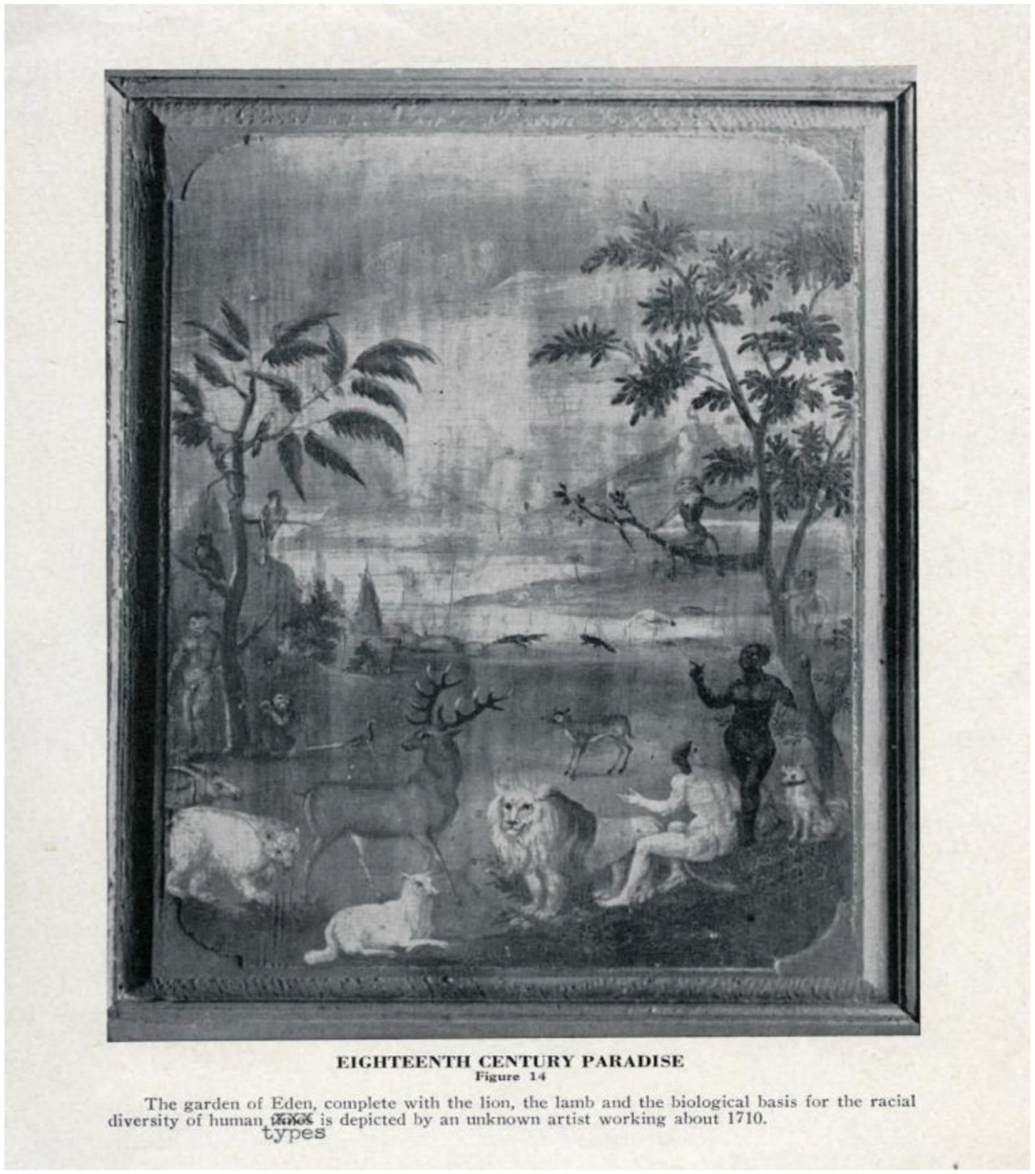
**Painting from around 1710, on a door-panel of the *Alte Apotheke* in southern Germany's Calow's pharmacy (Stern and Belar, 1953)**.

Yet, acceptable of the other, even tolerance, was not unheard of. Karl F. Gärtner (1722–1850) was a plant-breeder in the town of Calow in Southern Germany, acknowledged by Gregor Mendel in his foundational article of 1865. My post-doctoral adviser, Curt Stern, published in 1953 in the *Journal of Heredity* a note calling attention to a painting by one of the Gärtner's forefathers of around 1710, on a door-panel of Calow's pharmacy, the *Alte Apotheke*, of Paradise. It depicts the peaceful lion and the lamb with other animals and various plants, and of course, Adam and Eve: Adam is a white man; Eve is a black woman (Figure 1). Stern noted:

It seems that the pious unknown artist of the century of Rationalism – or was it his patron? – had contemplated upon the existence of different races of mankind. Must not their origins go back to the stem parents? Thus, the painter placed into Paradise a member of each of the most strikingly different human varieties to be the progenitors of the still existing diversity (Stern and Belar, 1953) ^1^.

However, not all humans were as tolerant to skin-color differences as the Calow painter. Stephen Jay Gould in the Introduction to his influential book *The Mismeasure of Man* traced *biological determinism* to the Platonian version of dialectics.

It holds that shared behavioral norms, and the social and economic differences between human groups – primarily races, classes, and sexes – arise from inherited, inborn distinctions and that society, in this sense, is an accurate reflection of biology (Gould, 1981, p. 20).

As I see it, it is the inadequate distinction between genotype and phenotype that is to be blamed for the blunder! *Biological* determinism is a relatively modern term, and *scientific racism* is, oddly enough, to a great extent a consequence or a product of the Age of Enlightenment and the establishment of the social notions of human equality. It is in such a context that the frame of mind toward Jews must be examined.

Contrary to physics and chemistry that became rational and determinist, already in the sixteenth and the eighteenth century respectively, it was only toward the nineteenth century that life-scientists like Linnaeus and Buffon, Blumenbach and Lamarck adopted reductionist principles to the analysis of the apparently purpose-directed phenomena of living beings and became essentially teleomechanistics (see Lenoir, 1982) ^2^. Finally, toward the mid-nineteenth-century, with the publication of Darwin's *Origin of Species* in 1859, biologists acquired a sound and strong foundation for a rational and determinist reductive science of biology.

Race, a loose term of socio-morphological classification (and evaluation/discrimination) of living creatures (plants included) now became a formal term in the sequence of the biological hierarchy of nature. But, whereas *species* were the ultimate assemblage of the Linnaean systematics for which an empiric criterion of discrimination could be conceived, namely the possibility of producing (fertile) hybrids between its members, *races* were an added—subjective—non-biological level of classification within species, between the members of which fertile hybrids may be produced. There have been no accepted empiric criteria to differentially classify any human races or other sub-species. Thus, how can genetics help decide who is a Jew?

## Who is a jew?

An ongoing dispute is: Are the Jews a religious community, a socio-cultural entity, an ethnic-biological classification, or what? The discordance is wide and has affected also Israel's court rooms. The leading article of the Israeli daily *Ha'aretz*, of Friday, 4 October 2013, is deploring **The Defeat of Israeliship**.

The refusal of the Supreme Court to accept the plea of 21 citizens [most of them well-known veterans of movements of civil rights], to recognize them as belonging to an Israeli nation […] is another expression of the failure of the civil struggle for the image of Israel. Sixty-five years after its establishment the authorities do not recognize an Israeli nation, disconnected of a religious definition or ethnic belonging. […]

In their decision, the judges deny the existence of an Israeli nation, and assert that it has not been proven that an Israeli nationality disconnected of a religious definition or ethnic belonging exists. […]

Fifteen years earlier, in 1998, journalist Michael Sheshar interviewed two retired Israeli Supreme Court judges asking “Who is a Jew?” (*Yedion Irgun Olei Merkas Europa* 139, August–September 1998):

Judge Haim Cohen responded: “the definition must be given by every single Jew for himself. If a person says of himself that he is a Jew, for me he is a Jew. This is his autonomy and nobody can decide for him or instead of him whether he is a Jew or not. There is no need in definitions.” Retired judge Menahem Alon emphatically contested this argument. He relied on the decision of the Knesset concerning the Law of Return: “The definition of the concept Jew, in this context, is: ‘He who was born to a Jewish mother, or converted, and does not belong to another religion’. This is the lawful definition in the State of Israel. And in my view this law is most essential. Otherwise we have no Jewish nation!”

In a later radio interview Justice Cohen said: “Judaism is a matter of religion for one and culture for another. I completely ignore the genes and biology. I respect the spirit that I received from my parents, and from my parents' parents.”

In their recent book, *Jews and Words*, the Israeli author Amos Oz and his daughter Fania Oz-Salzberger, insisted that it has been the Hebrew language that formed the thread which kept Jews together across the generations: “Jewish continuity has always hinged on uttered and written words, … Ours is not a bloodline but a textline” (Oz and Oz-Salzberger, 2014, p. 1).

The author of a recent book on *The Myth of the Jewish Race: A Biologist's Point of View*, Alain F. Corcos and his family managed to escape in time from the jaws of the pro-Nazi anti-Semitic Vichy regime in southern France in 1944. Following a detailed analysis of the history of the Jews and Jew hatred, the author summarized: “Many Jewish and non-Jewish writers find it difficult to accept the idea that Judaism is simply a religion and that Jews who abandon the faith, […] are no longer Jews” (Corcos, 2005, p. 18).

Other scholars reject such socio-cultural “superficial” definitions. For Solomon Zeitlin, Professor of Post Biblical Literature at Dropsie College, Jewishness is an inherited immanent socio-cultural property. Any allusions to material or political connotations are disparaging. According to him,

anyone who is born of a Jewish mother or one who embraced Judaism, regardless of whether he observes or does not observe the precepts is a Jew. Judaism is a universal religion and no one can exclude himself. The Jews are also united by their history and to a great degree by Hebrew culture. Since Judaism represents the genius of one people there is also the ethnic element which unites them. […] The land of Israel is not only the cradle of Judaism but Judaism as we know it today was molded there. Throughout the ages the Jews of the Diaspora longed for establishment of a messianic kingdom in the land of Israel. […] If Israel should become an ordinary, democratic industrial state it would be a great tragedy for Jewry and humanity as a whole” (Zeitlin, 1959, pp. 269–270).

These quotations of, and references to scholars and judges are only few of the eclectic definitions of Jewishness: Is Jewishness a religious-cultural property, a national-citizenship issue, or perhaps an ethnic-racial quality? It seemed that only the increasingly dominant authoritative status of “Science” in Western society would be able to provide an answer.

Researchers in the experimental sciences looked for, or endowed prominence to, so called immanent objective criteria of Jewishness, namely to ethnic genetic evidence. These started with claims of Jewish facial features, through specific frequencies of the polymorphic blood types, to the identification of so-called Jewish alleles of genes related to diseases, such as Tay-Sachs syndrome and cystic fibrosis among Ashkenazi Jews, or glucose-6-phosphate dehydrogenase deficiency among communities of Near-Eastern Jews, and more recently to claims of the identification of specific DNA haplotype sequences for Kohanim and for Levites Y-chromosomes ^3^.

## From emancipation to anti-semitism

The Age of Enlightenment in the second half of the eighteenth century, and the introduction of universal human values seemed to open the way for the emancipation of the Jews. Yet Jews have been, and still are conspicuously an inherently outstanding different presence within Western Society. Contrary to the discrimination against Blacks, who were still commonly considered to belong *biologically* to a different *race* or *species* than Whites (disregarding the evidence to the contrary of fertile hybrids), thus justifying their status almost as that of farm-animals, Jews were “others,” presumably belonging to a different religio-cultural human isolate that was conspicuously distinct from the gentiles among whom they lived.

However, the ancient Jew-hatred did not disappear at the Age of Enlightenment. The (relative) relaxation of the socio-cultural persecution against Jews was now replaced by *determinist biological* arguments for Jew-hatred, even though the borders between the spheres were vague, as evidenced, for example, by the interchange of biological type and national identity in King James Bible translating the Hebrew Cushite (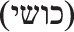) as Ethiopean.

As I conceive it, tragically it was largely the *socio-cultural* emancipation of the Jews that de facto instigated the *biological* discrimination against Jews. It must, however, be said that Jewish authors were among the first to suggest the biological distinctiveness of the Jews (see also Efron, 2013).

The German philosopher Johann Gottfried Herder (1744–1803) created the notion of “nation,” or *Volk*, as a meaningful entity, distinguished by landscape, climate, language, tradition, foreign intercourse, and also by heredity. Thus, in the first half of the nineteenth century the idea of the *Volk* became increasingly loaded with essentialist patriotic notions largely colored with biologist nuances. Even if the *identifying* properties of the race were in the realm of culture, language, or religion, still, it was argued, that their *essences* were biological by definition.

Herder, however, refused to adhere to a rigid racial theory, writing that “notwithstanding the varieties of the human form, there is but one and the same species of man throughout the whole earth.” He conceived of Jewry as an example of a community of individuals of national character, maintained by a religious and traditional culture, rather than race. Half a century later, Moses Hess (1812–1875), a close associate of Karl Marx and Friedrich Engels, became one of the earliest among the Jews who, disappointed with their emancipation, explicitly called for their national revival in Palestine. In his book *Rome and Jerusalem* (1862) he noted that “Jews are first of all a race.” More in terms of Herder's *Volk*, than those of “social-Darwinism” which was not yet conceived, Hess called for the Jews to reestablish their Jerusalem just as the Italians, under the leadership of Mazzini, established their Rome (Avineri, 1985).

Charles Darwin's (1809–1882) *The Origin of Species* of 1859 finally provided a material determinist means for life on Earth. It was soon interpreted in social determinist terms by persons like Herbert Spencer (1820–1903) in Britain and Ernst Haeckel (1834–1919) in Germany. Socio-political notions were increasingly interpreted biologically, in terms of hereditary inequalities among human beings. Thus, when the religious and cultural arguments for segregation and persecution of Jews lost power, biological claims for persecution held sway: Jews were of a different “race,” their specific traits were part of their biological essence.

By the 1870s and 1880s the claims that Jews belonged to a race that could be discerned in terms of the natural sciences, were repeatedly brought up, and the traditional hatred against them became increasingly physical in character. Against this background, by the end of the century, the plight of the Jews became ever more a political issue. Contrary to many of the assimilated or integrated Jews of the Age of Enlightenment and the Age of Romanticism, who concentrated on the cultural aspect of being Jewish, the Zionists-to-be stressed that Jews were not merely members of a cultural or a religious entity, but were an integral biological entity, even though they had been dispersed and had no country of their own. In other words, when the Zionists adopted the concept of *Volk* in terms of a nation-race, they claimed a different meaning to Jewishness than the centuries-long claims that the Jewish people were a distinct religious socio-cultural entity, rather than a biological entity. Moreover, even the *Halacha*, the strict Jewish law, which (technically) defines a Jew as a person born to a Jewish mother, also appended: “or one appropriately converted.” Thus, although discrimination of Jews had prevailed for almost two millennia, Jewish identity became “biological” only in the last decades of the nineteenth century. The term anti-Semitism was coined in the 1870s by the German publicist Wilhelm Marr (1819–1904). Anti-Semitism conceived the *socio-cultural* traits of Jews to be a consequence of their *biological* essence. Jew-hatred became *racism:* hatred of the Semitic race, *anti-Semitism*; it endowed biological justification to socio-cultural discrimination.

Marr's material reductionist Darwinian philosophy was explicit: “Anyone who cannot hold his own has to go” (Zimmermann, 1986, p. 67). However, the notion that the biological differences between people are responsible for their social differences spread exponentially in the twentieth century; it achieved, of course, its most catastrophic manifestation in Nazi Germany.

## Genetics of the jews

The insistence on the biological identity of the Jews, and the search for the phylogenetic relation of present-day Jewish communities to each other and to the ancient people of the Land of Israel, always applying the most updated scientific techniques, became a common obsession among Israeli and non-Israeli researchers.

The Jewish-British physician-virologist and eugenicist Redcliffe Nathan Salaman (1874–1955) was one of the first to examine the implications of the young science of genetics to Jews. Already in 1911, in the first volume of the *Journal of Genetics*, he published a paper entitled “Heredity and the Jews” (Salaman, 1911). In this paper Salaman tried to examine the distinct biology of the Jews with the new tools of Mendelian inheritance, which provided the basis for modern hereditary theory:

The object of this paper is to lay before anthropologists some results in the domain of Ethnology which, though arrived at by methods as yet foreign to anthropological research, promise a rich harvest in every direction. Mendelian methods […] have for the last decade been the all-powerful weapons of the modern student of heredity (Salaman, 1911, p. 273).

**Figure 2:**
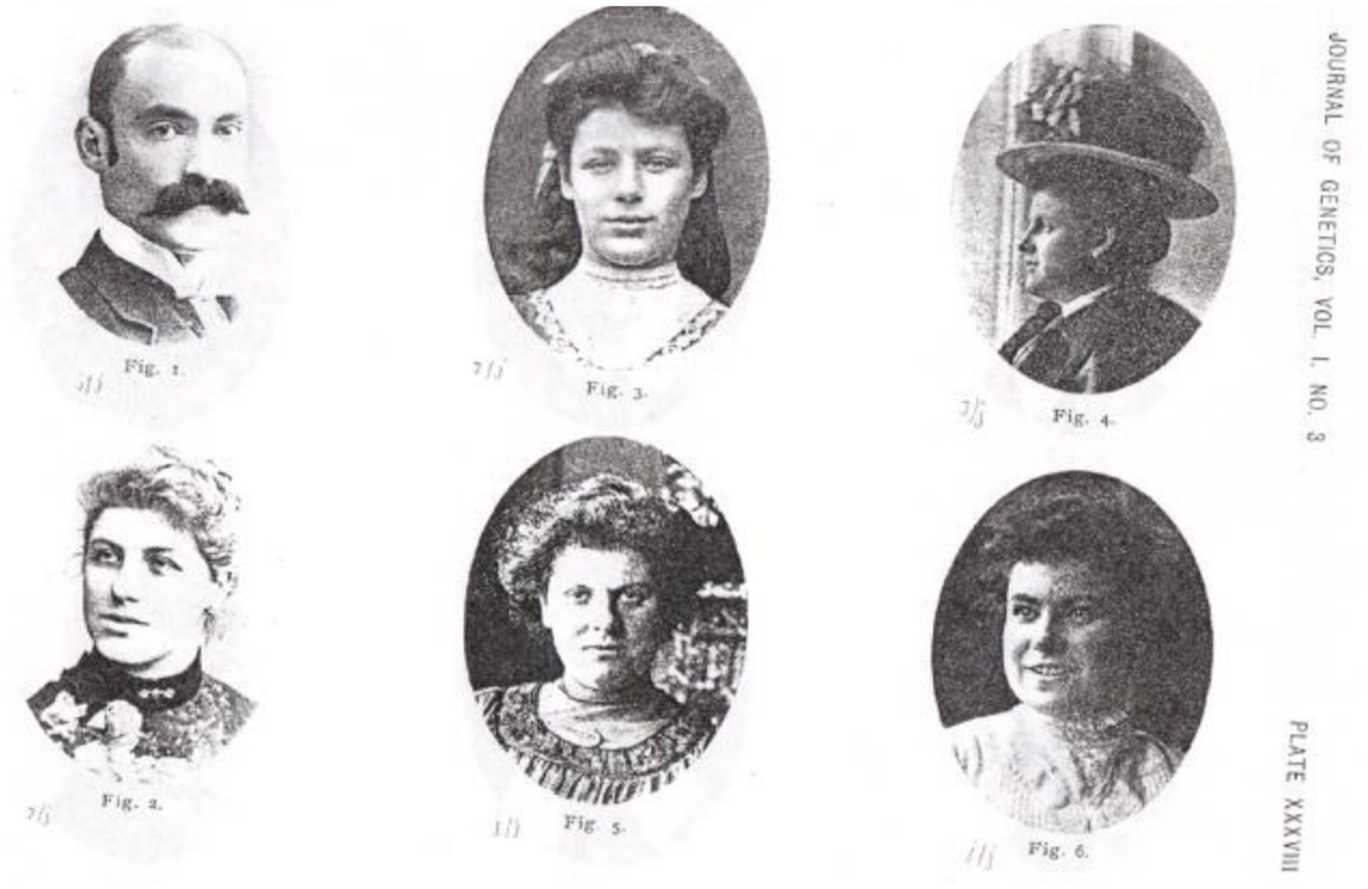
**A Jew (1) married to the daughter of Jew and Gentile (2) who gave birth to “Non-Jewish looking daughters,” (3, 4) and “Jewish looking children” (5, 6) (Salaman, 1911)**.

Salaman put special emphasis on the claim that Jews comprised a coherent biological entity. He pointed out that “Ethnologists may be said to agree that the Jew is not racially pure, but on the other hand […] the Jews constitute a definable people in something more than a political sense, and that they possess though not a uniform, still a distinguishable type” (Salaman, 1911, p. 278). Since Jews vary with respect to color, cephalic index and stature as any other population, “Jews cannot be defined according to any of these standards. There is, however, one characteristic which rarely escapes attention, and that is the Jewish facial expression” (Salaman, 1911–1912, p. 190). A Jew, according to Salaman, may be recognized by his facial features. With the help of “unbiased judges,” Salaman classified the progeny of 136 families of intermarriage between Jews and Gentiles. The progeny of these families were classified into 328 “Gentiles,” 26 “Jews,” and 8 “intermediates.” Among the progeny of 13 families of intermarriage of a “hybrid” and a Jew/ess (“backcrosses” in the genetic terminology) there were 15 “Jews” and 17 “Gentiles,” i.e., a good approximation to a 1:1 ratio (Figure 2). Thus, Salaman suggested that Jewishness is inherited and may be reduced to a single Mendelian factor, where the Jewish allele is recessive to the Gentile one (Salaman, 1911, pp. 281–285). In other words, the Jewish type has a solid biological basis, resting on the most advanced scientific achievements of the time. For Salaman Jewishness was a biological property.

How deep-rooted were the prejudices concerning Jews may be appreciated by juxtaposing Salaman's claim with Blumenbach's so-called liberal view, half a century earlier, in 1865, who held that whereas differences in human appearance were conditioned by climate and diet, Jews were an exception to the rules of nature: “the nation of the Jews who under every climate remain the same as far as the fundamental configuration of [the] face goes, [are] remarkable for racial character almost universal, which can be distinguished at the first glance even by those little skilled in physiognomy” (Blumenbach, cited by Efron, 2013, p. 903).

## Becoming molecular

The science of genetics became increasingly determinist toward a climax at mid-twentieth century, on the one hand it turned to probabilistic population genetics and on the other it adopted the physical model of the double helix of the chemically defined deoxyribonucleic acid molecules, and reduced heredity to sequences of nucleotides.

**Figure 3:**
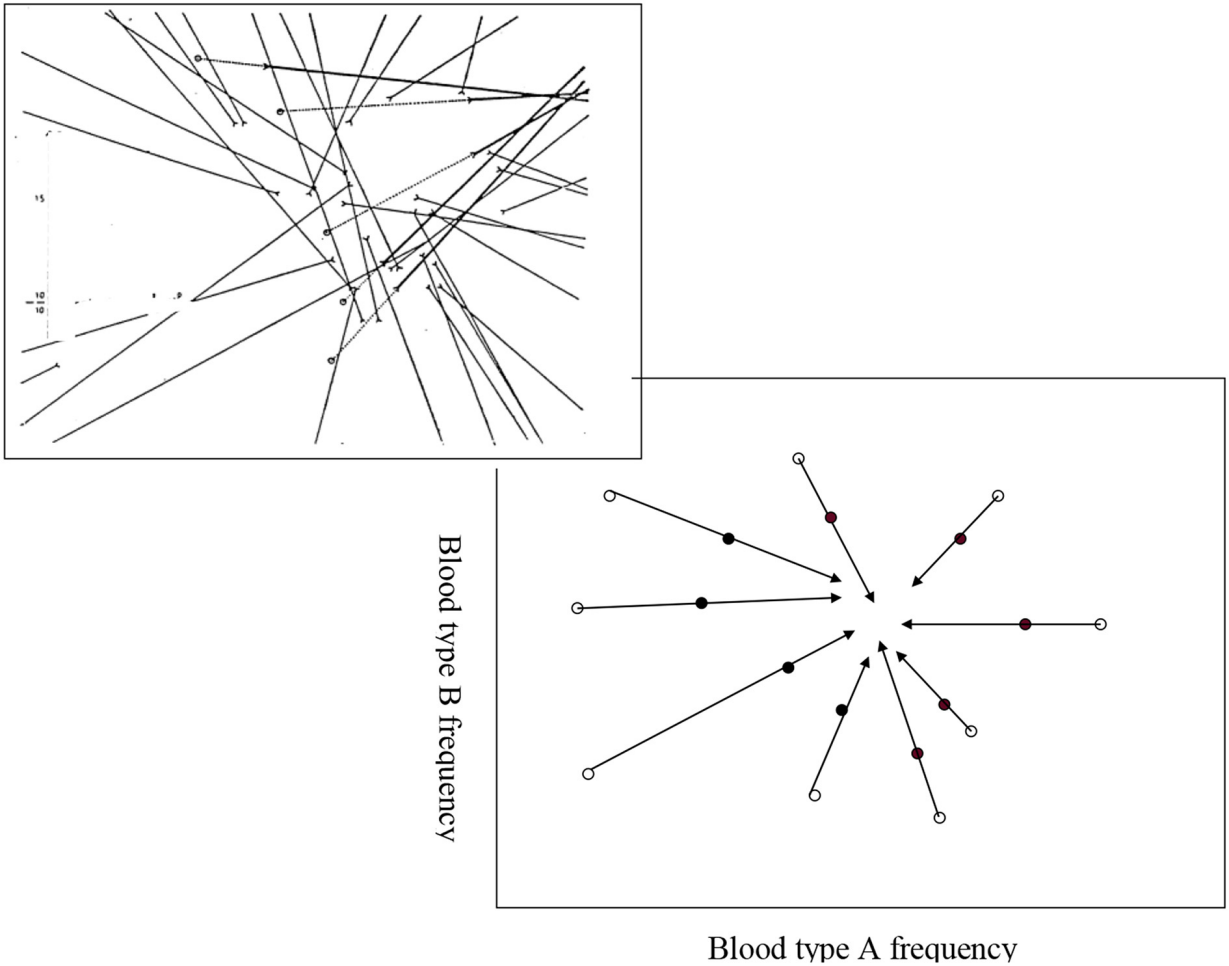
**Muhsam's attempt to identify the frequencies of the ABO blood type of the Jewish forefathers: vectors from “gentile environments” (open circles) to the corresponding “genuine” Jewish *eidoth* (closed circles)**. Right-lower, expected model; left-upper, observed.

Developments of research methods, especially those of blood typing, soon indicated to the extensive genetic polymorphism of human populations. Many efforts were made to find “typical” Jewish blood-type combination, and phylogenetic kinships between geographically and culturally close and distinct Jewish communities. These studies were summarized in 1978 by Mourant and colleagues in *The Genetics of the Jews* (Mourant et al., 1978). Efforts to deduce from such studies converging blood group frequencies of the hypothetical ancient Jews were not successful, yet as a rule, they did not discourage the authors from claiming for the reality of communities of progeny of common ancestry (see, e.g., Muhsam, 1964, and Figure 3).

Attempts to mobilize markers such as finger-print patterns to differentially characterize members of Jewish communities (e.g., Sachs and Bat-Miriam, 1957) failed likewise. More success, however, was gained with the genetic distribution of specific disease in Jewish communities. Tay-Sachs disease and Cystic-Fibrosis were conceived as Ashkenazi diseases, whereas Glucose-6-Phosphate Dehydrogenase Deficiency and other diseases were common in Sephardi Jews, and so on a notable list of inborn errors of metabolism (see Goldschmidt, 1963).

Developments in research methods, and primarily in the possibility of examining polymorphisms at the level of proteins (Lewontin and Hubby, 1966), and starting at the mid-1970s also of RNA- and DNA-sequences, enabled the comparison of genetic relationships even where no discernible morphological, physiological or behavioral variation existed. Not less significant, during the 1980s it became possible to examine simultaneously polymorphism in a very large number of sites along the DNA sequences. Once again the presumed relationships among Jewish communities, as well as their relation to non-Jewish communities were examined.

**Figure 4:**
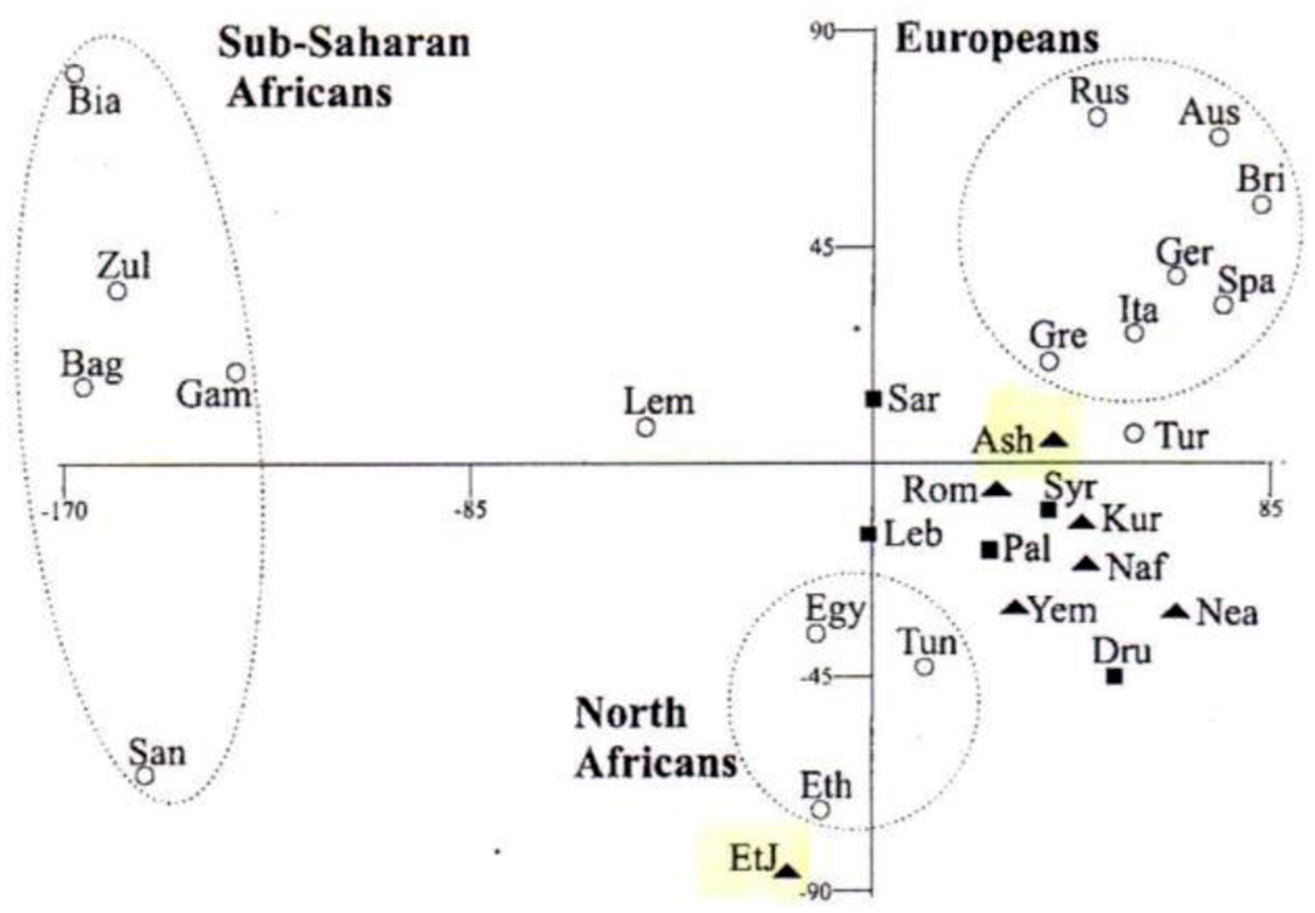
**Multivariant analysis of genetic variants of various populations, based on Y-chromosome hapolotype data (Hammer et al., 2000)**. Solid triangles represent Jewish populations, solid squares represent Middle Eastern populations.

The advances in analyses of DNA sequences provided the detailed specific nucleotide sequences of many individuals, and using algorithms of the most probable common forefathers *on the assumption of branching phylogenies* indicated to common progenitors of diverse Jewish communities and also to considerable overlap with those of Mediterranean populations (see, for example, Figure 4).

All these studies sampled Jewish and non-Jewish individuals. But how did they sample them? What were the criteria for Jewishness of the sampled individuals? This in itself is a moot issue that may crucially affect the conclusions.

These models of Darwinian evolution interpreted into *vertical* phylogenies are, of course, in agreement with the traditional Jewish historical lore of the contemporary Jews being the direct progeny of the historic residents of the Land of Israel. However, it is important to realize that the same genetic relationships may also result when considerable secondary *horizontal* associations, of intermarriages between communities of common culture, religion, or mere common domicile took place.

Although it makes sense that horizontal, intercommunity matings were, as a rule, less frequent than intra-community matings that maintained the vertical branching pattern, there is considerable historical evidence for inter-community mating, at the individual levels (Rabbis invited to serve in far communities, travelers, and emissaries sent to collect money in foreign communities, etc.), as well as at the level of whole communities: Historian Shlomo Sand (2009) and many others brings evidence of extensive community-wide proselytizing events, from North-Africa all the way to Southern Russia.

## The sons of aaron

One of the most exciting variables that were brought up and that became pivotal in the interpretation of phylogenetic data was that of the genetics of the Jewish priests. According to the biblical story, the tribe of Levi was destined for priesthood, and male-descendents of Aaron, the brother of Moses, were anointed as priests or Cohanim (pl. of Cohen). Thus, in the Jewish traditional patroclinous society, all persons with this name and its derivatives are vertical linear male-progeny of Aaron. If indeed, the tradition of the Cohanim was maintained, it makes sense to look for a common denominator among all these male progeny of Aaron, and if they furnish a model of marriage patterns for their communities their cohesion may provide an important indication for the common vertical roots of all Jews.

Already early on, in 1911 Salaman tried to get support from the Cohanim in his attempt to identity the “unmistakably Jewish expression”:

At this point one might with advantage consider the relation which the existence of the *Kohanim* as to the question of Jewish type. […] no Kohen, according to Jewish law, can marry a stranger, a proselyte or the daughter of the proselyte, or a divorcée: so that we have a sect whose descent may be regarded as strictly Jewish (Salaman, 1911, p. 279).

Obviously, Salaman did not succeed to identify any Jewish priests' phenotypic marker, not to mention genotypic ones. With the means at his disposal it was impossible to establish the Jewish-priest relations of the persons examined. He had to admit that “If now we review the physiognomies of the various *Kohanim*, it will be found that they exhibit no type in any way distinct from the other Jews.” Also the East-European physician and anthropologist Samuel Weissenberg (1867–1928) tried to rely on the tradition of the male-dynasty of “Aaronides (Kohanim) and Levites.” Some of these families keep centuries-old albums and seek to marry only with irreproachable families. Disappointedly, Weissenberg too found that the Aaronides and Levites represent, on the whole, the same type as the common Jews. “From these results it would be fundamentally incorrect to draw the conclusion that today's East European Jews are direct descendants of the ancient Israelites” (Efron, 1994, endnote 61, pp. 201–202).

Now, with the development of methods to follow specific DNA sequences of the human genome, interest in the Cohanim (and Levites) has gained new momentum as an instrument for proof of the common origins of the current Jewish ethnic-groups in the population of the Land of Israel two thousand years ago, as narrated in the biblical story (Skorecki et al., 1997).

The methodological breakthrough was obtained by Skorecki and coworkers, taking advantage of the differential chromosomal segregation pattern at cell-division in males and females. Of the 23 chromosome pairs of humans, one pair is different in females and males: whereas females have two copies of the X-chromosome, males carry one X-chromosome, like that of females, and its smaller partner, the Y-chromosome. Females contribute one X-chromosome (like any of the other chromosomes, called autosomes) to each progeny; males contribute an X-chromosome to half of their progeny and a Y-chromosome to the other half of their progeny. Progeny who obtained two X-chromosomes are females; those who inherited one X-chromosome and a Y-chromosome are males. Thus, following a marker linked to the Y-chromosome may point to a biological lineage leading back to an ancient common male-progenitor. If indeed priesthood has been maintained by strictly following the patrilineal tradition, then all Cohanim should carry the derivatives of the priest Aaron's Y-chromosome. *Derivatives*, rather than the original sequence, because obviously, rare mutations have occurred in its nucleotide-sequence over the millennia. Since mutations are rare events, each mutation would probably be specific and unique, and the frequency it is encountered would be proportional to the number of generations passed since its occurrence. Furthermore, when the role of the Y-chromosome is largely reduced to that of a “mechanical” counterpart of the X-chromosome in providing orderly chromosome segregation, most sequences on the Y-chromosome are considered to be of rather little relevance to natural selection, and the abundance of a mutation on the Y-chromosomes may accordingly be used as a reliable indicator of its age.

Of course, these Y-chromosomes need not be restricted to Cohanim, but being faithfully transferred from one Cohen male to another, it may suggest the construction of a pedigree tree all the way back converging on Aaron the Priest.

Although the Y-chromosome is the smallest human chromosome, its DNA molecule is 57 million base-pairs (Mb) long and its non-recombinant region, which is the region that satisfies the biological criteria for ancestry analysis, is 24 Mb in length (of which only less than half remain after filtering procedures). Thus, as a rule only choice-segments along the chromosome are selected for study. Such segments, some thousands of base-pairs long, may be looked at in concert, *haplotypes*, ^4^ which provide a unique combination of polymorphisms along a Y-chromosome, and represent the whole chromosome.

[…] we sought and found clear differences in the frequency of Y-chromosome haplotypes between Jewish priests and their lay counterparts. Remarkably, the difference is observable in both the Ashkenazic and Sephardic populations, despite the geographical separation of the two communities. […]

We identified six haplotypes […]. Applying the χ^2^ test to the frequencies of the Y-chromosome haplotypes distinguishes priests from the lay population. […]

We further identified subjects as being of Ashkenazic or Sephardic origin. […] the same haplotype distinction can be made between priests and lay members within each population. This result is consistent with an origin for the Jewish priesthood antedating the division of world Jewry into Ashkenazic and Sephardic communities (Skorecki et al., 1997).

With due respect to the always important reductive simplifying assumptions made, this time it appeared that the efforts bore fruit: Molecular markers were found that indicated common denominators which were significantly more common among the Y-chromosomes of the Cohanim than Israelites. No less important, these denominators were common in Sephardi as well as Ashkenazi Cohanim. The social and political, also the religious meaning of a biological continuity, of “we are all Jews,” often mentioned or implied, now attained overt corroboration, at least as far as the Cohanim represented a fair sample of Jews. *The Guardian* of January 2, 1997 reported:

Researchers in genetics confirmed today something that was a holy scripture in Israel for 3300 years.

They examined the Y-chromosome of Jewish Kohanim and found that, indeed, they vary from those of the Jewish people. […]

Although, according to tradition, all 14 million Jews in the world are the children of Abraham, the molecular biologists find difficulties in reconstructing the biblical links. Two distinct Jewish populations, Ashkenazi and Sephardi, of different even though somewhat blurred genetic composition, exist […]

[Researchers] found that in certain respects Kohanim in the different communities vary from the rest of their respective ethnic-groups and are more similar to each other. The studies confirm that their chromosomes may be calibrated as a genetic “clock” of father-to-son […] and also supports an ancient religious tradition. The Jewish priesthood appears indeed to have been founded by a single ancestor […]

Mainly two types of polymorphisms were followed: that of *microsatellites*, a type of repetitive DNA, the number of repeats varying due to (intra-chromosomal) recombination between sequences, which may occur in up to 1/1000 cell divisions, and *single nucleotide polymorphisms* (SNPs), due to mutations that are orders of magnitude rarer. Already some years earlier, a rather high correlation was found between Y-chromosome haplotypes of Ashkenazi and Sephardi Jews that exceeded even that between (non-Jewish) Mediterranean groups (Santachiara et al., 1992). The authors suggested that if people of common origin diversified and became Sephardi or Ashkenazi respectively, this diversification probably affected mainly morphological markers (structural discernible characteristics) that were of value in the respective specific circumstances that the different communities were exposed to, rather than molecular differences, such as Y-chromosome polymorphisms, that were neutral in processes of selection.

Contrary to the data of the main body of non-Cohanim Jews, which indicate to considerable blending with their non-Jewish neighbors, the variation of the Y-chromosome of the Cohanim is mainly limited to derivatives (by unequal recombination and mutation) of a single prevalent haplotype—the Cohen Modal Haplotype, or CMH. It is convincingly prevalent among Sephardi (61%) as well as Ashkenazi Cohanim (69%). Among non-Cohanim Israelites (i.e., non-Cohanim Jews) the CMH comprises only some 0.1 per cent, suggesting “gene migration” from Cohanim to other Israelites (Thomas et al., 1998). “Given the relative homogeneity of Cohen Y-chromosomes in comparison with those of the Israelites we can conclude definitively that adoption of the status has not occurred on a very large scale over a long period of time. [Furthermore,] making an educated guess as to what the ancestral chromosome was and then calculating the distance from the current chromosomes to this imagined ancestral one” places the age of the one or few common progenitors some 3000 years ago. Indeed, given the assumptions and educated guesses, and failing to take into account all other possible scenarios, these results “appeared to be a striking confirmation of the oral tradition” (see Goldstein, 2008, pp. 30–38).

However, not all data accorded with these findings. Uzi Ritte, of the Department of Genetics at the Hebrew University, examined the number of specific Y-chromosome haplotypes among Jewish persons of Sephardi origins carrying the name Cohen (9) and among lay Jewish persons of the same communities (90). The corresponding number for Levites of Iraqi origins were 7 and 110, respectively. He concluded that there was no unusual clustering of Y-haplotypes among Cohanim, compared to that among lay Israelites (Ritte, personal letter).

Still, the tradition of following discrete genetic markers on the one hand, and the development of methods for following a large number of variables at the level of DNA sequences, together with the development of sophisticated computational methods for the detection of the interconnections between them on the other, provided researchers a renewed opportunity to examine historical claims, or to perform “genetic archeology,” in spite of inherent difficulties. Goldstein summarizes:

Our studies of the Cohanim established that present day Ashkenazi and Sephardi Cohanim are more genetically similar to one another than they are to either Israelites or non-Jews.

Among the Cohanim we see greatly reduced diversity, and the Cohanim Y-chromosome is a subset of what is seen among Israelites (Goldstein, 2008, p. 65).

The apparent achievement of the children of the priest Aaron in maintaining their distinct status over a very long time and across very diverse socio-geographic distances is even more remarkable, when juxtaposed with that of the remaining children of the tribe of Levi, the Levites.

No haplotype frequently common to Levites was found. But among the Ashkenazi Levites a cluster of haplotypes with a very high degree of relatedness was found. The R-M17 Y-chromosome haplogroup, is rare in Israelite Jewish populations (<5%), and generally rare or absent in populations of the Near East. It is, however, prevalent in Belarusians (50%) and the Slavic-speaking Sorbs (66%). Apparently only Ashkenazi Levites, and not the rest of their fellow Jews, have received a significant male contribution of Slavic origin. Data suggest that the R-M17 chromosomes were transmitted horizontally to Ashkenazi Levites (or the other way round) relatively late, somewhere between the fourth and eleventh century.

Considering such findings, it made sense to assume that the strong forces that acted to preserve the single variable—Y-chromosome transmitted from father to son—was relevant also to Israelites, though less so than with respect to Cohanim and Levites. Consequently, the similarity of Y-chromosome markers is consistent with the claim of Middle-Eastern paternal origin of present day Cohanim (Thomas et al., 1998; Behar et al., 2003), and may indicate similar conclusions with respect to that of Jews in general.

However, is it sensible to draw similar conclusions with respect to the Ashkenazi ethnic-group from, say, the clusters of haplotypes of the Ashkenazi Levite? Behar and associates point out that the Levite cluster of R-M17 haplotype is very common in non-Jewish populations of north-east Europe. Thus, isn't it reasonable to assume that the origin of these haplotypes among Levites (and other non-Levite Jews) is in *horizontal* transmission, namely that male progeny of some non-Jewish Europeans who intermarried with a Jewess (inadvertently) acquired the status of Levites?

### The khazar connection

Although there is no much enthusiasm among many historians to the assumption that Asian Khazars or some other Europeans were involved in the origin of the Ashkenazi, it is not possible to exclude an important input of such founders to present-day Ashkenazi (Behar et al., 2003, p. 777). The possibility of the existence of the Levites' cluster in the Middle-East even before the segregation of the Jews from the rest of the nations of the Middle-East is rejected by Behar et al. (2004): The differences in mutation rates and elimination rates by random drift of SNPs and of microsatellites, respectively appears to be enough to explain many of the apparently conflicting findings concerning the relationship among *eidoth* of common origin.

Thus, Behar and associates accept with satisfaction the fact that research of the biological foundation of the Jews and their inter-relationships became an instrument for clarifying the Jewish identity in the process of the Zionist project of ’ingathering of exiles’ in Israel:

The comparative study of patterns of NRY [Non-recombining Region of the Y-chromosome] variation among Ashkenazi Jews and other populations has revealed evidence for an unexpected and unusual historical event, which was not appreciated using other, more conventional historical approaches. This finding may motivate historians and social scientists to seek further information regarding the possibility of such an event and, more generally, to include information gleaned from studies of DNA variation in the repertoire of tools used to uncover historical events of interest (Behar et al., 2003, p. 778).

Can DNA-sequence analyses exclude claims that Ashkenazi Jews stem mainly from the conversion of the Inner-Asian Khazars at the second half of the first millennium, rather than from West-European Jews of Middle-Eastern origins?

David Goldstein is more explicit:

Could Khazaria, I wonder to this day, be the source of Ashkenazi Levite R-M17 Y chromosome?

As with much else of genetic history, there is no way to be sure. […]

I was initially quite dismissive of Koestler's identification of the Khazars as the “thirteenth tribe” and the origin of the Ashkenazi Jewry. Was this not just another self-aggrandizing Lost Tribe narrative bereft of evidence?

I am no longer so sure. The Khazar connection seems no more far-fetched than the spectacular continuity of the Cohen line or the apparent presence of Jewish genetic signatures in a South African Bantu people. […] I cannot claim the evidence proves a Khazari connection. But it does raise the possibility, and I confess that, although I cannot prove it yet the idea does now seem to me plausible, if not likely (Goldstein, 2008, pp. 73–74).

Advancements in molecular methods that allows today large scale whole genome screening, say of single nuclear polymorphisms (SNPs), and access to the local populations of southern Russia and the Caucasus and Caspian Sea states, stimulated new search for possible imprints such as those of Khazar history.

Working mostly with autosomal chromosomes, one may expect to find regions that are “identical by descent” (IBD) that is passed by inheritance even after several generations (when they would be shorter due to recombination). So, if inter-community marriages occurred, it may be possible to see, say, Iraqi Jews with IBD typical to Polish Jews. However, if such events happened so long ago that the IBD regions disappeared, inevitably indications of inter-community mating disappear.

Recent whole-genome DNA sequencing of modern Caucasus populations prompted Eran Elhaik to revisit the “Khazarian Hypothesis,” suggesting that Eastern European Jews descended from the Khazars, and compare it with the “Rhineland Hypothesis” that depicts Eastern European Jews as a “population isolate” that arrived in Eastern Europe roughly at the thirteenth and fifteenth centuries, and emerged from a small group of German Jews who migrated eastward and expanded rapidly (Elhaik, 2013).

The complete data set contained 1287 unrelated individuals of 8 Jewish and 74 non-Jewish populations, genotyped over 531,315 autosomal single nucleotide polymorphisms (SNPs). The author has applied a wide range of population genetic analyses to compare the two hypotheses and showed that a sole Judean ancestry cannot account for the vast population of Eastern European Jews in the beginning of the twentieth century without the major contribution of Judaized Khazars.

The findings support the hypothesis that posits that European Jews are comprised of Caucasus, European, and Middle Eastern ancestries, and portray the European Jewish genome as a mosaic of Caucasus, European, and Semitic ancestries, thereby consolidating previous contradictory reports of Jewish ancestry.

We conclude that the genome of European Jews is a tapestry of ancient populations including Judaized Khazars, Greco-Romans Jews, Mesopotamian Jews, and Judeans and that their population structure was formed in the Caucasus and the banks of the Volga with roots stretching to Canaan and the banks of the Jordan (Elhaik, 2013).

But the Khazar theory of Ashkenazi Jews depends primarily on the identification of the present-day progeny of the Khazars whose DNA-sequences may be sampled. Whereas Elhaik based his analyses on the populations of central and south Caucasus, Behar together with 30 coauthors (Behar et al., 2013) claimed that present-day progeny of the Khazar live north of the Caucasus. They find no indications of contemporary Ashkenazi Jews to be progeny of the Khazars. According to them the inhabitants of southern Caucasus, sampled by Elhaik, are related and stem from the countries further south, namely those of the orient; hence no wonder that Ashkenazi Jews carry DNA polymorphisms akin to them. The issue turns out to be more ethnographic, of population movements in time and space, rather than one of genetic haplotype variation phylogenesis.

Here again the risk of circularity of the argument is exposed: Geneticist determine the genotypic details of socio-ethnologists' classifications, whereas socio-demographers rely on geneticists findings to bolster their classifications.

## Horizontal vs. vertical kinships

It is important to keep in mind that most analyses of the phylogenies of the Jews are based on the assumption that the present polymorphisms reflect repeated events of a vertical, tree-like branching from common origins of human populations, which occurred at different and successive occasions. Allan Templeton reminded us that all such trees are nowadays accessible in computer programs, *designed* to provide the best possible *vertical trees* that genetic data offer (Templeton, 1998, 2008). However, Templeton presents the trellis model, according to which there was also a constant *lateral* or *horizontal* flow of genes in human populations intertwined with that of vertical evolution. An increasing number of investigators support the model according to which human populations are entangled more like a woven cloth than an ordered mosaic pattern. Theories are by principle underdetermined, and it would be impossible to exclude one or the other also in the future (Gannett, 2004, p. 330, see also Weiss and Long, 2009).

With the advances in analyses of DNA sequences, allowing the identification of detailed specific sequences of individuals, indications lead to sequences of common progenitors of many Jewish communities and also to a considerable overlap with Mediterranean populations. If interpreted into *vertical* phylogenies these inevitably support the traditional Jewish historical lore of the contemporary Jews being the direct progeny of the historic residents of the Land of Israel. The same genetic relationships may, however, also indicate secondary *horizontal* associations, of intermarriages between communities of common culture, religion, or mere common domicile.

Already in the late 1990s did Ritte and his associates (Ritte et al., 1993a,b) try to analyze simultaneously the inheritance of patrocliuous (Y-chromosome) DNA sequences and matroclinuous mitochondrial DNA (mtDNA) sequences in representatives of six Jewish communities (Ashkenazic, North African, Near Eastern, Yemenite, Minor Asian/Balkanian, and Ethiopian). They concluded that “communities, whose haplotypes are mostly Caucasian, are more closely related; significant differences that were found among some of them possibly indicate the effects of admixture with neighboring communities of non-Jews” (Ritte et al., 1993a).

**Figure 5:**
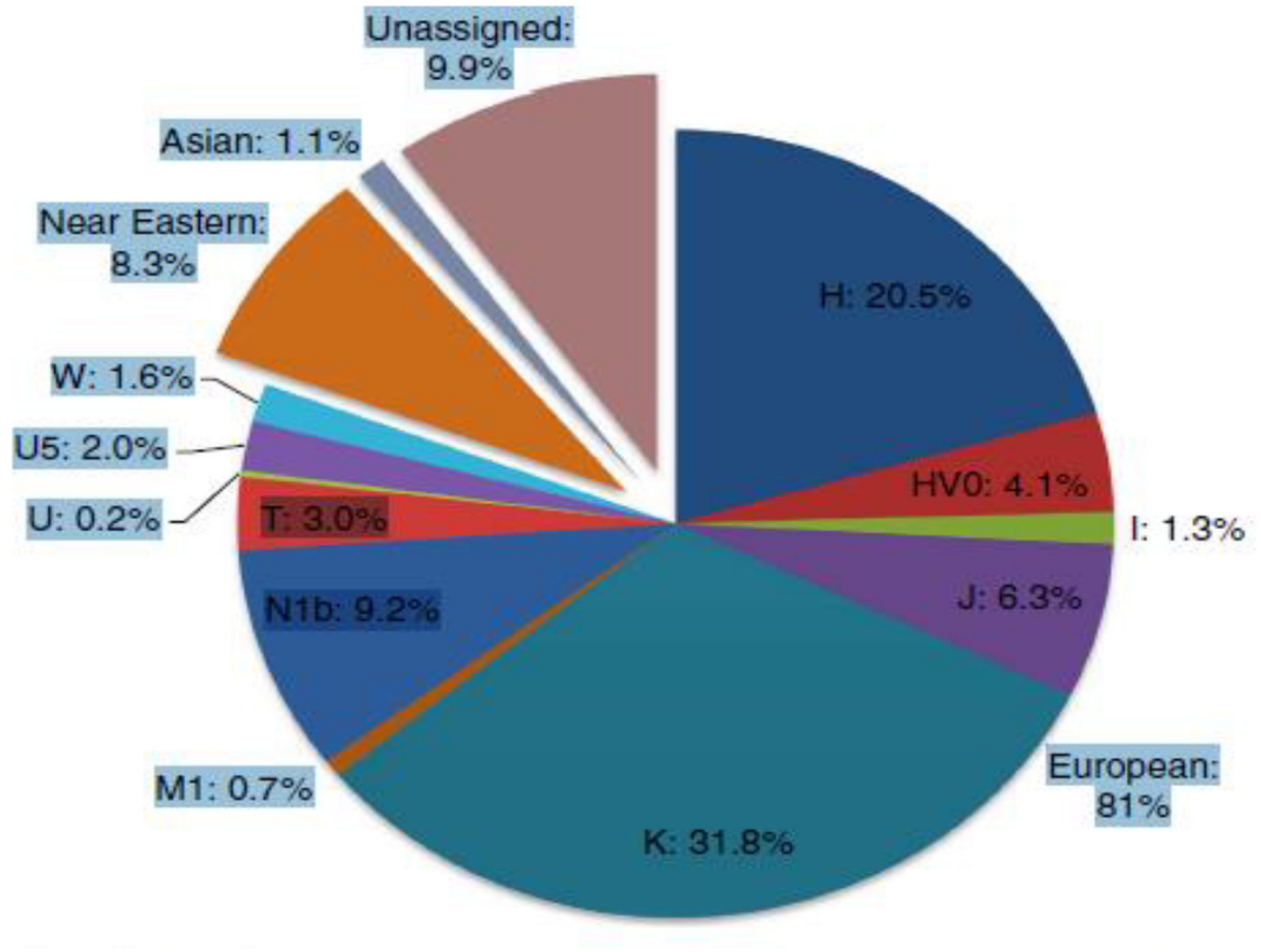
**Estimated contributions of European mtDNA lineages to the Ashkenazi mtDNA pool shown by major haplogroup (Costa et al., 2013)**.

More recent studies of the distribution of mitochondrial DNA (mtDNA) which, like Judaism is passed along the maternal line, indicate that Ashkenazi mtDNA is highly distinctive, with four major and numerous minor founders. All four founders, ~40% of Ashkenazi mtDNA variation, have ancestry in prehistoric Europe, rather than the Near East or Caucasus. Furthermore, most of the remaining minor founders, share a similar deep European ancestry. Thus, the great majority of Ashkenazi maternal lineages are assimilated within Europe (Figure 5). These results point to a significant role for horizontal phylogeneses due to the conversion of women in the formation of Ashkenazi communities in prehistoric Europe (Costa et al., 2013).

Whereas on the male side there may have been a significant Near Eastern (and possibly east European/Caucasian) components in Ashkenazi ancestry, the maternal lineage mainly trace back to prehistoric Western Europe (Costa et al., 2013, p. 2).

Overall, it seems that at least 80% of Ashkenazi maternal ancestry is due to assimilation of mtDNAs indigenous to Europe, most likely through conversion (Costa et al., 2013, p. 8).

**Figure 6:**
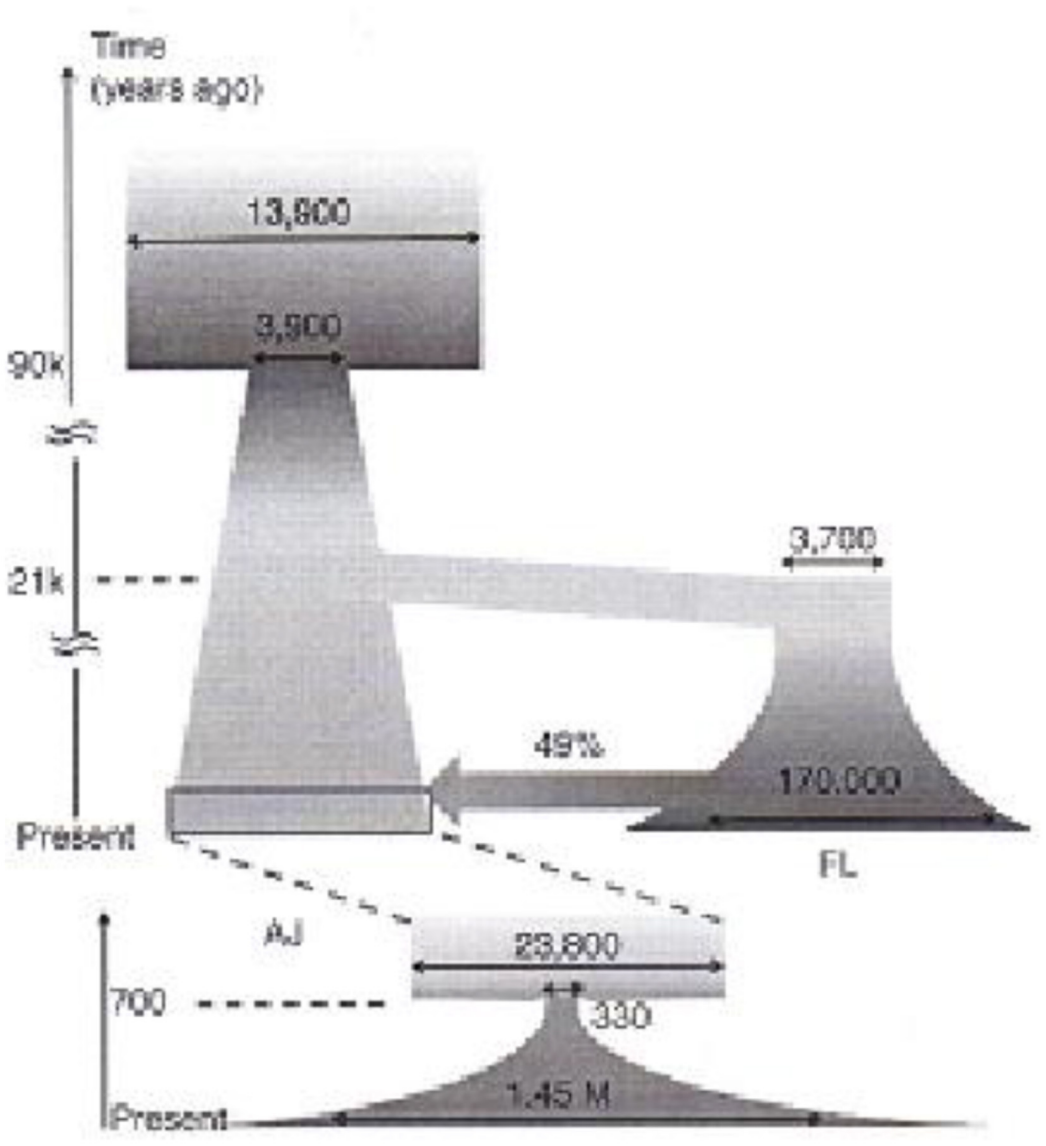
**Reconstruction of the Ashkenazi Jewish (AJ) and European (FL) demographic history**. The wide arrow represents an admixture event. Horizontal arrows: effective population sizes (Carmi et al., 2014).

Current advances in laboratory techniques together with sophisticated computational analyses allowed high-depth sequencing of 128 complete genomes of Ashkenazi Jews (AJ), compared with European (FL) samples of nuclear SNP arrays. These went even further in reconstructing a two dimensional picture of the “demographic history” of the AJ (Figure 6) (Carmi et al., 2014). By applying the most advanced computation methods Shai Carmi and colleagues integrate, besides the vertical generation changes, also the impact of horizontal factors on the evolution of genomes such as vectors of population-size bottlenecks and periods of intensive trans-population admixture. Not surprisingly, Ashkenazi Jews prove to compose a distinct yet quite integral branch of European genomic tapestry.

## Coda

Jews were recognized over the ages as a People of a distinct religion, or as a People with unique socio-cultural bonds, in various contexts and at different times. But it was claimed that what ultimately maintained the Jews identity were their genealogical linkage: Jews were perceived as the descendants of Abraham, Isaac, and Jacob, the three patriarchs, not only spiritually but primarily biologically.

Obviously, what kept Jews identity were their language, culture, tradition and religion. Thus, whatever their biological hereditary kinships, both the trans-generational vertical, and intra-generation horizontal relationships are secondary consequences. However, the increasing reliance on scientific reductionism in biological thinking of the last two centuries eventually culminated in turning the evidence of DNA sequences into the *essence* of the characterization of Jewishness rather than its *consequence*. Still, in spite of repeated efforts, there is no agreed upon criterion to identify Jews, and samples examined for the distribution of biological or molecular markers all depend on the preconceived biases of the investigators. Races, it is assumed, may differ in inherent properties that are evaluated differentially. But races are not biological-meaningful classification entities. And if so, why is racism a bad property? The answer must be: Because it provides socio-cultural justifications for discrimination on the basis of presumed and irrelevant biological properties.

### Conflict of interest statement

The author declares that the research was conducted in the absence of any commercial or financial relationships that could be construed as a potential conflict of interest.

## Footnotes

^1^ The typed correction was made in the original reprint I obtained from the authors.

^2^ Teleomechanics: The adoption of reductive empirical principles of the experimental sciences in the analysis of the apparently purpose-directed phenomena of living beings (see Falk, 2013).

^3^ It is noteworthy that even the Nazis, after mobilizing the most advanced means and methods of science of their time for identifying Jews, reverted to using the yellow star patch attached to the garment as the identifying device.

^4^ Haplotype, a contraction of the phrase “haploid genotype,” is a set of closely linked genetic markers, a combination of DNA sequences, at adjacent locations (loci) present on one chromosome, which tend to be inherited together (not easily separable by recombination).
